# Preparing and assessing the physiochemical properties of curcumin niosomes and evaluating their cytotoxicity in 3T3 and MCF-7 cell lines

**DOI:** 10.22038/AJP.2021.18163

**Published:** 2021

**Authors:** Narges Ashraf Ganjooei, Mandana Ohadi, Seyyed Mohammad Amin Mostafavi, Behzad Behnam, Abbas Pardakhty

**Affiliations:** 1 *Pharmaceutics Research Center, Institute of Neuropharmacology, Kerman University of Medical Sciences, Kerman, Iran*; 2 *Herbal and Traditional Medicines Research Center, Kerman University of Medical Sciences, Kerman, Iran*; 3 *Department of Pharmaceutical Biotechnology, Faculty of Pharmacy, Kerman University of Medical Sciences, Kerman, Iran*

**Keywords:** Niosome, Curcumin, Cytotoxicity, MTT, Cancer, Drug delivery

## Abstract

**Objective:**

Application of vesicular drug delivery systems has made major progress in pharmaceutical science and technology. Niosomal drug delivery is potentially efficient to improve the pharmacokinetic and pharmacological properties of many compounds. Curcumin (CUR) has several documented anticancer activities; however, it has a low bioavailability that necessitates the development of efficient delivery systems. Accordingly, different niosomal preparations were prepared and evaluated in the present study to find a suitable delivery system.

**Materials and Methods:**

Span and Tween 20, 40, 60, and 80 were employed with various concentrations of cholesterol for studying the ability to form curcumin-loaded niosomes. Multiple characterization techniques including visual evaluation, particle size analysis, stability, encapsulation efficiency (EE), and release profile were studied. Cytotoxicity of curcumin niosomes on MCF-7 and 3T3 cell lines was determined using MTT assay.

**Results:**

Visual and particle size analysis indicated the formation of seven niosomal formulations in the micron size range, while the formulation consisted of Tween 40/cholesterol (50/50 M%) with 0.05% w/v CUR had an average diameter of 475 nm. The latter formulation was selected and it had EE of 78.5%. The CUR release profile showed 18.7% release over a period of 300 min. The MTT results showed that CUR incorporation significantly increased the cytotoxicity of niosomes and the extent of toxicity was higher in MCF-7 cells.

**Conclusion:**

In this study, a simple niosomal formulation was developed for CUR loading with favorable physicochemical properties. The presented niosomal curcumin had also considerable effects in cell toxicity studies, which can be suggested for future anticancer studies.

## Introduction

Curcuma longa L. rhizome has been employed as a spice in Asia for many years and Curcumin (CUR) as the yellow pigment present in turmeric possesses antitumor activity indicated by multiple documented *in vitro* and *in vivo* studies (Patel et al., 2020 ▶; Afshari et al., 2021 ▶). Curcumin has been investigated in multiple inflammatory diseases, metabolic disorders, and various cancers (Prasad et al., 2014 ▶; Shakeri et al., 2019 ▶). CUR possibly interacts with transcription factors, tumor necrosis factor alpha (TNFα), tumor suppressor genes, and enzymatic systems associated with carcinomas, such as cyclooxygenases and lipoxygenases (Sarkar et al., 2016 ▶). However, poor aqueous solubility, limited bioavailability, and fast metabolism in humans are the major challenges of CUR administration for cancer treatment (Anand et al., 2007 ▶). To overcome these problems, techniques to effectively deliver CUR with new platforms, such as liposomes, Noisome, carbon nanostructures, and various nanoparticles have been developed (Rahimi et al., 2016 ▶; Akbari et al., 2020 ▶; Moballegh-Nasery et al., 2020 ▶; Rezayi et al., 2020 ▶; Slika and Patra, 2020 ▶; Mahmoudi et al., 2021 ▶). Niosomes are surfactant-based vesicles usually represented by a bilayer structure and are generated by two major components, including nonionic surfactants and stabilizing additives. They have different sizes, with biodegradable nature and less toxic properties, and have been applied as drug delivery system (DDS) for many pharmaceuticals (Pardakhty and Moazeni, 2013 ▶). Niosomes have been studied as an effective DDS, especially for agents with limited stability, poor solubility, or rapid degradation (Amoabediny et al., 2018 ▶; Akbari et al., 2020 ▶). In this regard, Qi Xu et al. (Xu et al., 2016 ▶) prepared a CUR-loaded niosome system that made the CUR soluble in the lipophilic bilayer and introduced it as an acceptable carrier for CUR. In another recent study, Sahab-Negah et al. (Sahab-Negah et al., 2020 ▶), designed a novel CUR -loaded niosome nanoparticle and evaluated the CUR effect on human glioblastoma stem-like cells. To the best of our knowledge, there are few studies for the evaluation of niosomal curcumin and it is necessary to investigate newer formulations. Our main objective was to synthesize new CUR-loaded niosomes while investigating their physicochemical properties. In addition, the cytotoxic effect of CUR-niosomes was assessed on MCF-7 and 3T3 cells.

## Materials and Methods

**Preparing niosome formulations**

Niosomes were prepared using the thin-film hydration method which was described in detailed in our previous report (Behnam et al., 2018 ▶). In brief, sorbitan monostearate (Span 40), Tween 40, and cholesterol (chol) (Sigma, Germany) were weighted and dissolved in chloroform with different molar ratios as shown in Table 1. The final lipid concentration was set to 300 µM and formulations were nominated from A to M (Table 1). The rotary evaporator (EYELA, Japan) was used to evaporate the organic solvent to form a thin film in a round-bottom flask at 65°C, which was then rehydrated after 24 hr using deionized water at a pH of 7.4. Next, for obtaining the smaller niosomes, the hydrated niosomes were sonicated (sonopuls HD- 3200, Germany) with a frequency of 20 kHz for 5 minutes. For obtaining curcumin niosome (CUR-Niosome) formulations, CUR (Sigma, USA) was dissolved in the lipid phase at a 0.05% w/v ratio. Moreover, for the selected formulation, CUR-Niosome was filtered at 50°C through membrane filters (0.45 µm pore size) for three times and 0.22 µm filter pore size (Jet Biofil, Korea) for three times. 

**Table 1:** Different formulations of proposed niosomes. Niosome formation (+), absence of niosome formation (-), not evaluated (NE).

Cholesterol (M%)			Surfactants (1:1 molar ratio)
C) -	B) +	A) -	Tween 20/Span 20
F) +	E) +	D) +	Span 40/Tween 40
I) +	H) +	G) +	Span 60/Tween 60
L) -	K) -	J) -	Span80/Tween 80
M) +	NE	NE	Tween 40

**Characterization of niosomes**

Through the Zeta-sizer Nano ZS (Malvern Instruments Ltd., UK), the niosomes average size was evaluated using a helium-neon laser at 630 nm and room temperature. In brief, niosomal specimens were properly diluted (1:10) with deionized water and their size was assessed according to the dynamic light scattering (DLS) technique (Sahab-Negah et al., 2020 ▶). In addition, size evaluation was done through VASCO™ particle size analyzer (Cordouan, France) for the selected smaller niosome formulations and each experiment was carried out in triplicate. We also assessed the CUR-niosome morphology through direct observation 48 hr after synthesis in a light microscopic (Olympus DP71). For assessing the stability, the selected niosome formulation was kept in the refrigerator (5°C) and size distribution analyzing was done at the intervals of 1 week and 1, 3 and 6 months. 

**Drug encapsulation efficiency (EE%)**

Encapsulation efficacy of CUR in niosomes was calculated through sedimentation of niosomes at 18000 × g for 30 min. Then the sedimented niosomes were lysed with isopropyl alcohol to extract the entrapped curcumin. The EE% was calculated via dividing the amount of entrapped CUR by the amount of total curcumin (in the supernatant and within niosomes). CUR level was measured through the UV-Vis spectrophotometry (Optizen 3220 UV) at 420 nm (Kavousi et al., 2018 ▶). 

***In vitro*** **drug release**

Vertical glass Franz-type diffusion cell (active surface area: 3.46 cm^2^; receptor phase volume: 15 ml) was applied for *in vitro *drug release of CUR from niosomes at 37°C and pH of 7.4 for 300 minutes using the membrane (Visking tube, MW cut-off 12,000 D) as the barrier between donor compartment and receptor compartment. The receptor section was filled with deionized water and ethanol (50:50 v/v%) and the donor compartment with CUR-niosomes (1 ml). Then, at set intervals, 1 ml of the samples was withdrawn from the receptor replaced with the equal volume of fresh receptor medium followed by measuring the permeated CUR level (Samarehfekri et al., 2020 ▶). 

**Cytotoxicity studies**

In this study, MCF-7 cell line (human breast tumor) and 3T3 cell line (mouse fibroblast) were obtained from the Pasteur Institute, Iran. Regarding cell development, we used DMEM/F12 medium which was prepared with penicillin (100 U/ml), streptomycin (100 µg/l), and FBS (10%) (biosera, Iran). The cells were allowed to proliferate in an incubator providing 5% CO_2 _at 37°C. Cytotoxicity studies were performed by seeding 10,000 cells in each well within a 96-well cell culture plate and incubating during 24 hr. The following day, after removal of the medium, different niosomal formulations were prepared in fresh medium (without FBS) and added to cells. Niosome formulations were serially diluted to obtain either empty niosomes with concentrations of 300 to 0.6 µM (nominated as: Nio 300, Nio 180, Nio 120, Nio 60, Nio 6, and Nio 0.6) and curcumin loaded niosomes which were nominated as: Nio 300-CUR 0.05, Nio 180-CUR 0.03, Nio 120-CUR 0.02, Nio 60-CUR 0.01, Nio 6-CUR 0.001 and Nio 0.6-CUR 0.0001). The prepared formulations were added to the cells and incubated for the next 24 hr. Subsequently, after adding MTT solution (5 mg/mL in PBS), the cells were incubated in darkness for 3 hr. Then, by removing the medium and adding dimethyl sulfoxide (DMSO), the absorbance underwent examination at 570 nm with a microplate reader. Moreover, the cell viability was determined as 100% for the untreated control cells. The experiments were carried out in triplicate in different day intervals. Moreover, the cell viability (%) was determined via the below formula (Ohadi et al., 2020 ▶; Ohadi et al., 2021 ▶): 

Cell Viability (%) = (OD of the treated group/OD of the control group) × 100

**Statistical analysis**

All data shown here were carried out in triplicate and were reported as mean±SD. The analysis of data was performed by GraphPad Prism 6 software (GraphPad Software Inc., CA, U.S.A) using ANOVA and Tukey post hoc test. The difference less than 0.05 was considered significant.

## Results

**Characterization of niosomes**

Niosome formation was evaluated simply through microscopic observations. As shown in Table 1, span 80/Tween 80 complex was not able to produce niosome at any cholesterol level. Moreover Tween 20/Span 20 surfactant mixture was able to make niosomes at only 40% cholesterol level of lipid film. 

**Figure 1.**
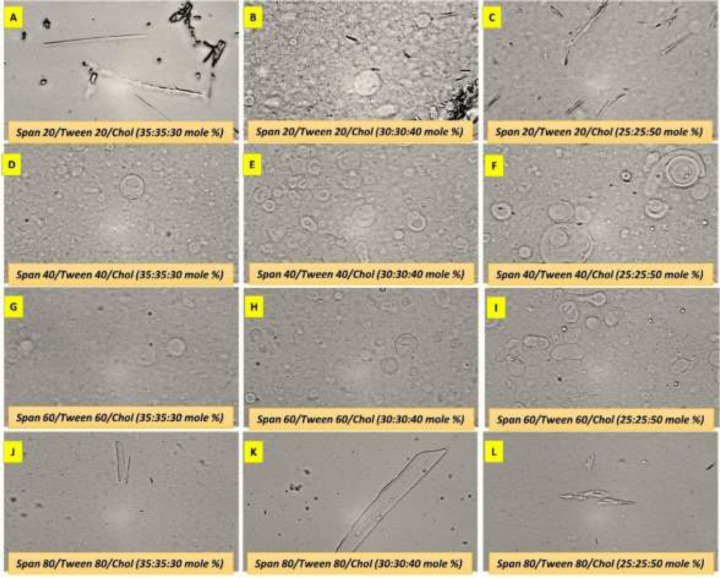
Light microscopic images of curcumin (0.05% w/v) niosomes. (Magnification is 400×)

Preparation of all other formulations led to the formation of niosomes that are shown in Figure 1. Niosome formation was also accompanied with the creation of a milky suspension of niosomes upon hydrating with water. Clearly, it is visible that large unilamellar vesicles (MLVs) and multi-lamellar vesicles (MLVs) are predominantly present in the niosomal formulations of B, D, E, F, G, H and I, however smaller niosomes are also present.

**Figure 2:**
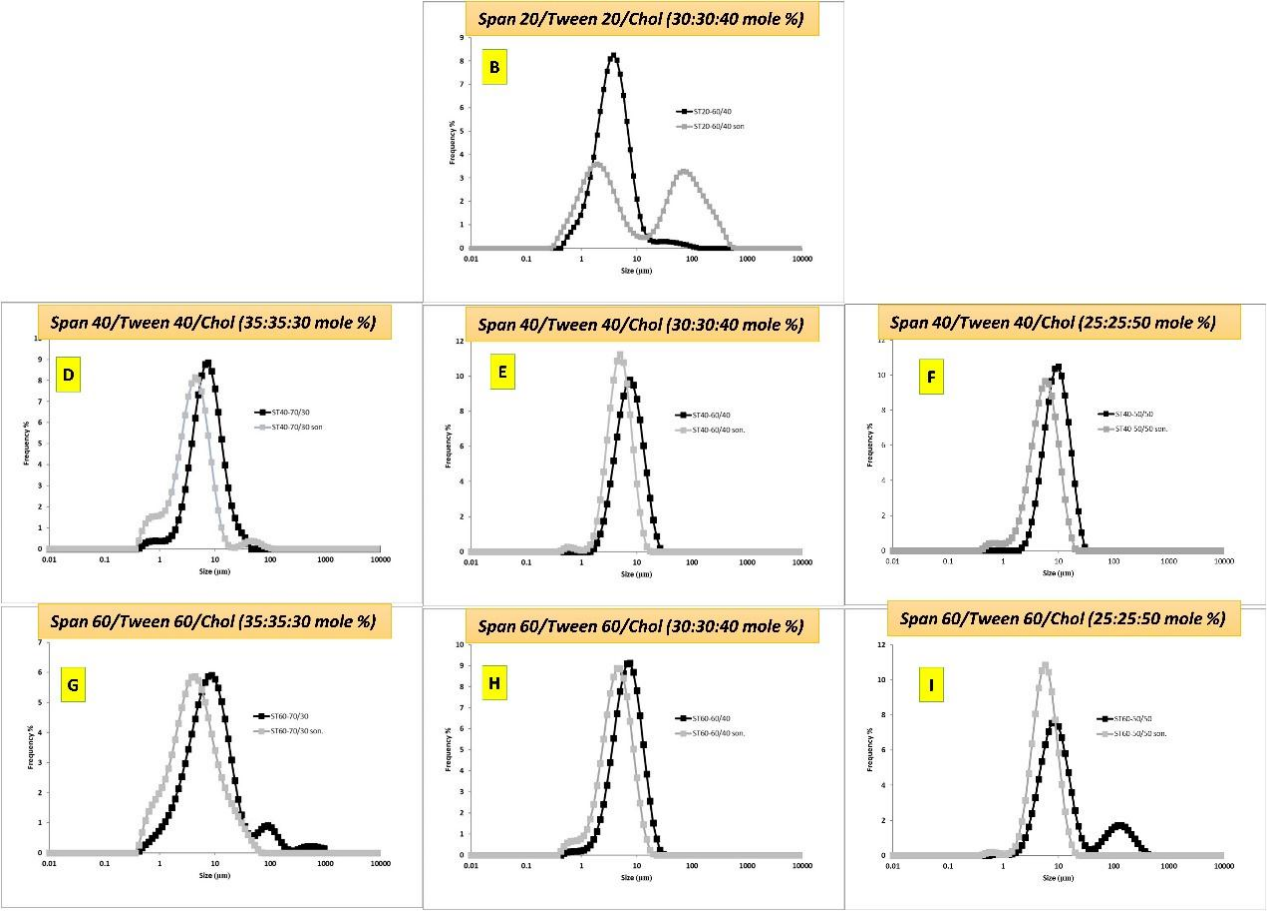
Size distribution of different curcumin (0.05% w/v) niosomes before and after sonication (son).

Light microscopic image of niosomes also revealed the polydispersity of vesicles. They had mostly spherical shape with a thick layer membrane. Formulations of A, C, J, K, and L did not lead to the formation of niosome and large particles and crystals of cholesterol were evident. The DLS assessment showed the particle size distribution characteristics of CUR-niosomes in Figure 2. The prepared niosomes were sonicated as described and the effect of sonication is also depicted besides the original plots (denoted as son in the figures). It is evident that sonication can lead to the reduction of size while it could lower the polydispersibility index. Interestingly, formulation B (‏Span 20/Tween 20/Chol (30:30:40 M%) ‎was not able to undergo the sonication process and creation of a new peak at larger sizes could be related to the formation of cholesterol crystals..

**Figure 3:**
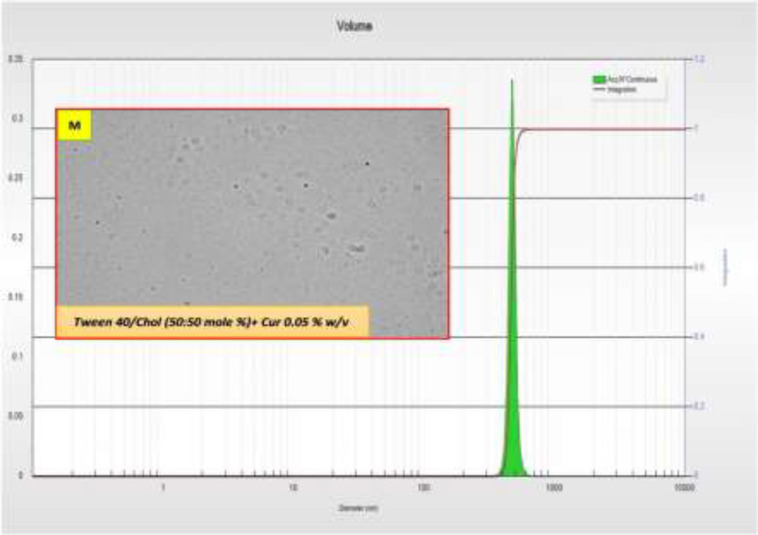
Photomicrograph of curcumin niosome ((Tween40/chol (50/50)-CUR 0.05) (inset), magnification is 400×) and related size distribution pattern

Size reduction was done through sonication, but still the niosomes were in the micron ranges and as we aimed to obtain nano-niosomes, formulation of M, consisted of Tween 40/chol (50/50) was chosen for further studies. This formulation was in the nanometer range without the need for sonication and as shown in figure 3, it has a mean diameter of 477 nm after two rounds of filtration through membrane filters. Tiny round niosomes are visible in microscopic image (Figure 3, inset). In addition, most of the niosomes were in a uniform distribution as revealed by the graph in Figure 3.

Storage stability of the selected formulation, i.e. M formulation (Tween40/chol (50/50)) was also evaluated. As shown in Table 2, the volume diameter of niosomes was not changed significantly and it was approximately remained in the nanometer range, while also the smaller niosome population was decreased. Encapsulation efficiency assessment was done as described earlier and the results showed the entrapment of 78.5±9% of curcumin molecules into Tween40/chol (50/50) niosomes.

**Table 2:** Storage stability of Tween40/chol (50/50)-CUR 0.05

Time	Volume diameter (d_v_), nm	Frequency (F%)
1 week	42.43	2
	477.36	96
1 month	57.43	7
	495.93	92
3 months	464.05	98
6 months	472.59	97

***In vitro*** **drug release profile**

**Figure 4:**
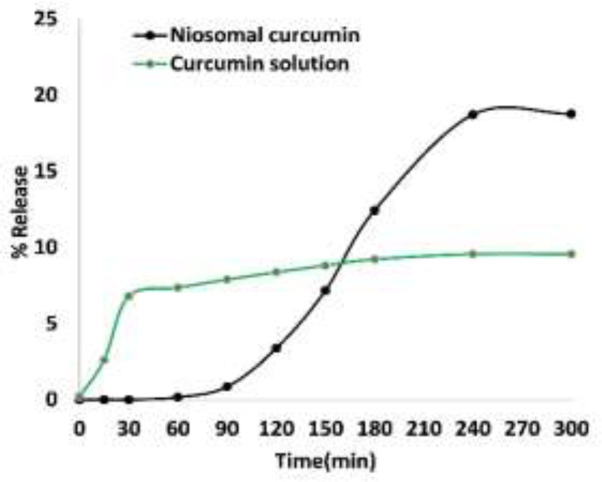
The release profiles of CUR-niosomes and curcumin solution

Figure 4 shows the *in vitro *release behavior of CUR-niosomes and curcumin solution. The results show that curcumin will not release from niosomes in noteworthy concentrations during the first 50 minutes. Afterwards, curcumin release continued up to 19% over 300 minutes. On the other hand, curcumin is released into the receiver phase from curcumin solution since the earliest minutes and subsequently it turns into a constant state. 

The slow release of curcumin from niosome particles can be attributed to its hydrophobicity and good interaction with niosome layers that can be retained and increases the therapeutic outcomes (Yallapu et al., 2013). Niosome is an appropriate drug carrier acting as a pool 

for releasing compounds in a steady, programmed, and stable fashion (Rathee et al., 2020 ▶).

**Cytotoxicity assay**

**Figure 5:**
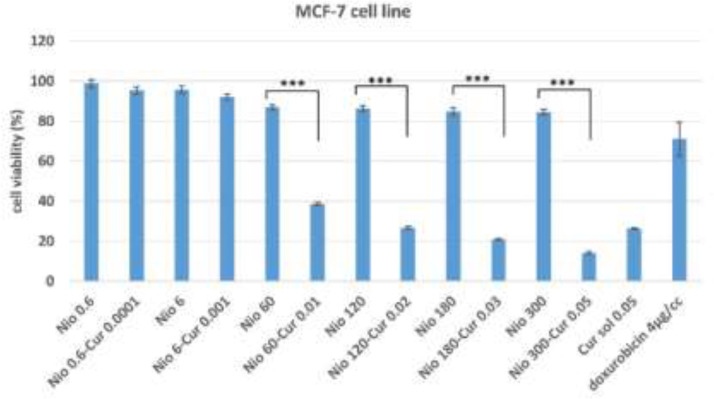
Cytotoxic effect of different concentrations of CUR-niosomes and niosome solutions on MCF-7 cells line. Data are presented as Mean±SD. *p< 0.05, **p< 0.01, ***p< 0.001

**Figure 6:**
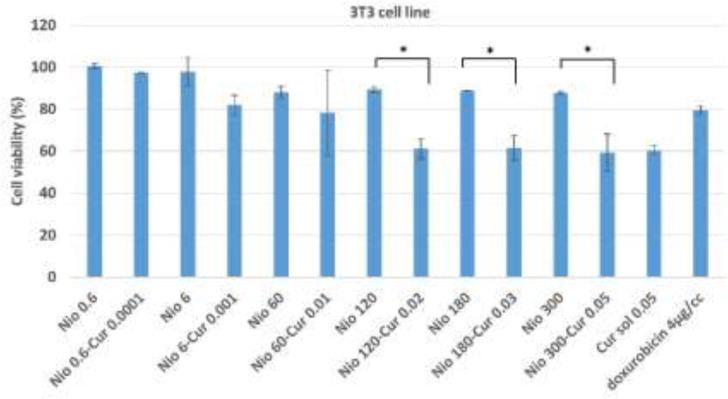
Cytotoxic effect of different concentrations of CUR-niosomes and niosome solutions on 3T3 cells line. Data are presented as Mean±SD. *p< 0.05, **p< 0.01, ***p< 0.001

The cytotoxicity of CUR -niosomes on MCF7 and 3T3 cells was assessed using MTT assay (Figure 5 and Figure 6). MTT assay is a sensitive technique for fast analysis of cell metabolic activity upon cell exposure to various biological molecules. Results show that regardless of the type of formulation, increasing the concentration led to the increased cytotoxicity, however in many groups this increase is negligible. Empty niosomes in both cells expressed a good cytotoxicity profile because all of their viability results are higher than 80%. Empty Nio 0.6 and Nio 6 are almost nontoxic as their viabilities are higher than 95%. Moreover, the highest concentration of niosomes could reduce the viability only up to 84% in MCF7 cells, while in this case, a high concentration of 300 µM of niosomes is present. The other important result that can be figured out from the plots is that curcumin incorporation into niosomes will decrease the viability of empty niosomes in both cells. While this reduction is not high for Nio 0.6-CUR 0.0001 and Nio 6-CUR 0.001, but all other groups showed at least 50% reduction in viability in MCF-7 cells (p value for all other niosomal groups were less than 0.0001). Similarly in 3T3 cells, curcumin loading into Nio 120, 180 and 300 led to significant reduction in cytotoxicity (p values are 0.034, 0.044, and 0.031 respectively). 

Other obvious finding is that cell toxicity is higher in MCF7 cell lines in most of the treated groups. The highest toxic formulation, i.e Nio 300-CUR 0.05 reduced the viability of MCF7 cells to 19% while this value was 59% in 3T3 cells.

## Discussion

In this study, we aimed to develop new formulations of curcumin based on non-Ionic surfactants. Therefore, different preparations of surfactants were employed to find the best formulation. Span and Tween 20, 40, 60, and 80 with varying concentrations of cholesterol were evaluated to make 12 formulations of A to L (Figure 1). Among the various formulations, span/tween 40 and 60 were the most efficacious surfactants to produce vesicular niosomes of curcumin as these compositions have been reported in different niosomal systems (Barani et al., 2019 ▶). In fact, during the evaluation of different active pharmaceuticals for incorporation into niosomal systems, it is possible that the proposed surfactant composition could not produce a stable niosomal system, even if that composition was good for other pharmaceuticals. For example, herein, span/tween 20 and 80 (except for the span20/tween20/chol 30:30:60)-CUR 0.05) were not able to produce niosomes at any cholesterol level while a recent report by Asadikaram et al. showed that this composition can be also favorable (Asadikaram et al., 2021 ▶). Therefore, it is essential to evaluate any of the proposed surfactant mixture in the presence of main pharmaceuticals for niosome formation and stability. We aimed to choose nanometer sized niosomes and therefore sonication was performed to produce smaller niosomes (Figure 2). However, more reduction in the size was not possible until removal of one of the surfactants and as visible in Figure 3, tiny niosomes were prepared through the Tween40/chol (50/50) (M formulation) composition. This formulation was chosen for future studies in this report. Comparable to our report, a recent remarkable study by Sahu et al. showed that CUR can play a similar role to cholesterol and can form niosomes with only Tween molecules (Sahu et al., 2020 ▶). In the study performed by Xu et al., they showed that the diameter of the CUR-niosomes were reduced with an increase in the homogenization pressure. Moreover, they reported the smaller diameter and zeta potential of the CUR-niosomes compared to blank niosomes since curcumin binds to the lipophilic part of the surfactant, leading to a denser CUR-niosome structure and lower particle size (Xu et al., 2016 ▶). Herein, EE was determined to be 78% and it is comparable to other recent niosomal CUR with EE of 87% (Span20/Tween20) to 95% (Span20/Tween20/Chol) as reported by Akbari et al. (Akbari et al., 2020 ▶) and 15% (Span60/Chol/PG) to 99% (Tween80/chol/PG) as reported by Shehata et al. (Shehata et al., 2021 ▶) for various niosomal preparations. This good encapsulation efficiency of curcumin can be associated with the highly lipophilic nature of CUR that resulted in the remarkable incorporation of CUR into niosome layers (Zhao et al., 2013 ▶). EE has been reported to be directly related to lipid concentrations, where higher lipids will lead to the higher loading of lipophilic CUR (Mohanty and Sahoo, 2017 ▶). Similarly, it was shown that EE will increase with the growing amount of cholesterol (Akbari et al., 2020 ▶). Storage stability is a key factor for designing pharmaceutical preparations and herein, CUR-niosomes (M formulation) maintain their primary size after 6 months in the range of ≈470-490 nm. Minor reduction in the population of smaller niosomes could be attributed to the formation of newer larger niosomes from the smaller one, however it did not make any effect on the overall size distribution and we can conclude that the prepared niosomes were stable during the studied period (Table 2). DDS candidates of specially chemotherapeutics must retain their active ingredient over time and release the drug gradually. Herein, *in vitro* curcumin release showed a progressively curcumin release in comparing to its solute state that quickly released CUR in early minutes (Figure 4). However, we did not analyze CUR release over a longer period of time. In the cell toxicity studies, we tried to understand the primary effects of various concentrations of M formulation on two cell lines. The data showed that curcumin incorporation into the proposed niosomes could greatly increase the cytotoxicity. Importantly, when considering lower concentrations of surfactants, the empty niosomes would not express high toxicity (Behnam et al., 2018 ▶). These two factors are favorable when considering a new DDS. Here, the best recommended dosage is for 120 µM of niosomes, in which the viability of MCF-7 cells are 86% and it can be reduced up to 26% (60% reduction) after curcumin integration. A question arises when looking at the curcumin solution where this form of drug has even high toxicity; however, it is lower than the niosomal form. It must be noted that curcumin has a poor solubility and its efficient delivery is of utmost importance. Beyond curcumin delivery and its protection from biological degradation, DDS of niosomes could also promote cellular uptake (Ojeda et al., 2016 ▶). Similarly, a recent report by Sahab et al. showed that intracellular CUR levels increased by niosome-mediated delivery in comparison with freely dispersed CUR (Sahab-Negah et al., 2020 ▶). Interestingly, the other favorable result of the present study was that the cytotoxicity of almost all formulations were higher in MCF-7 cells that are cancerous cell lines when compared to 3T3 cell lines that can be regarded nearly as normal cells. However, we did not examine the exact state of cells after treating with these formulations, and the rate of apoptosis and necrosis must be assessed in future studies. In upcoming studies, newer formulations considering different concentrations of cholesterol with Tween 40 and various concentrations of CUR could be hypothesized while the effects of other surfactants could also be evaluated. Moreover, the transdermal penetration potential of these niosomes could be evaluated as CUR has been studied in different topical applications (Mohanty and Sahoo, 2017 ▶).

Overall, the present study introduced a new formulation of niosomal curcumin that had favorable physicochemical properties. We observed that Tween 40/chol (50/50)-CUR 0.05 w/v formulation had a stable size (475 nm). The presented niosomes retained CUR with a high EE of 78% and released it slowly over time. Moreover, they showed good stability during 6 months of storage. Niosomal curcumin had higher toxicity to MCF-7 cell lines in comparison to 3T3 cells. The 120 µM concentration of niosomes could be applied in future studies as it has the least cytotoxicity while it was effective to significantly improve curcumin cytotoxicity in cancerous cells. 
